# Metformin Inhibits the IL-6-Induced Epithelial-Mesenchymal Transition and Lung Adenocarcinoma Growth and Metastasis

**DOI:** 10.1371/journal.pone.0095884

**Published:** 2014-04-30

**Authors:** Zhongquan Zhao, Xiaoming Cheng, Yubo Wang, Rui Han, Li Li, Tong Xiang, Luhang He, Haixia Long, Bo Zhu, Yong He

**Affiliations:** 1 Institute of Cancer, Xinqiao Hospital, Third Military Medical University, Chongqing, China; 2 Department of Oncology, Fuzhhou General Hospital of Nanjing Military Commond, Fuzhou, China; 3 Institute of Respiratory Diseases, Xinqiao Hospital, Third Military Medical University, Chongqing, China; 4 Department of Respiratory Medicine, Daping hospital, Third Military Medical University, Chongqing, China; 5 Biomedical Analysis Center, Third Military Medical University, Chongqing, China; H. Lee Moffitt Cancer Center & Research Institute, United States of America

## Abstract

**Objective:**

Epithelial-mesenchymal transition (EMT) plays an important role in cancer tumorigenesis. However, the underlying mechanisms of EMT in lung adenocarcinoma, and how this process might be inhibited, remain to be explored. This study investigated the role of IL-6 in lung adenocarcinoma cell EMT and explored the potential effects of metformin on this process.

**Methods:**

Invasion assay and MTT assay was performed to determine cell invasion and cell proliferation. Western blotting, immunofluorescence, real-time PCR, ELISA, and immunohistochemistry were performed to detect the expression of IL-6, E-cadherin, Vimentin, and p-STAT3.

**Results:**

We discovered that IL-6, via STAT3 phosphorylation, could promote lung adenocarcinoma cell invasion via EMT in vitro. This was supported by the inverse correlation between E-cadherin and IL-6 expression, positive correlation between IL-6 and vimentin mRNA expression and between STAT3 phosphorylation and IL-6 expression in tumor tissues. Importantly, metformin inhibited tumor growth and distant metastases in tumor-bearing nude mice and reversed IL-6-induced EMT both in vitro and in vivo. Furthermore, we found that blockade of STAT3 phosphorylation might be the underlying mechanism of metformin inhibition of IL-6-induced EMT.

**Conclusions:**

Collectively, our present results show that enhanced IL-6 expression, via STAT3 phosphorylation, is a mechanism of EMT in lung adenocarcinoma. We found that metformin could inhibit IL-6-induced EMT possibly by blocking STAT3 phosphorylation.

## Introduction

Non-small cell lung carcinoma (NSCLC) is a leading cause of cancer death worldwide, resulting in an overall 5-year survival rate of less than 15% [1]. Adenocarcinoma accounts for more than half of all NSCLC. Despite advances in treatment, cancer recurrence and formation of distant metastasis are still the main cause of death in patients with lung adenocarcinoma. A better understanding of the mechanisms underlying the formation of distant metastases is required to facilitate the development of effective therapeutic strategies for lung adenocarcinoma patients. Growing evidence reveals that epithelial-mesenchymal transition (EMT) plays a pivotal role in tumorigenesis, drug resistance, relapse and metastasis of various cancers [2]–[7]. Numerous reports have suggested that EMT is a marker of poor prognosis in patients with NSCLC [8]–[11]. Therefore, elucidating the mechanisms underlying EMT and identifying molecular targets and efficient drugs that inhibit this process is a promising approach to attenuate drug resistance, inhibit growth and reduce metastasis, thereby improving the overall survival rates of lung adenocarcinoma patients.

EMT is a complex process, involving dissolution of cell-cell junctions and loss of apical-basolateral polarity, resulting in transition of epithelial cells into migratory mesenchymal cells with invasive properties [12]. Migratory mesenchymal cells after transition are endowed with mesenchymal markers, such as vimentin and N-cadherin, but have lost epithelial markers such as E-cadherin and γ-catenin. Loss of E-cadherin expression is generally accepted as a hallmark of the EMT process [13]. During this process, various transcription factors, such as SNAIL, ZEB1, ZEB2, Twist and E2.2, are key controllers that repress E-cadherin expression. MicroRNAs (miRNAs), such as members of the miR-200 family, are also involved in EMT regulation by targeting the key transcription factors involved in direct repression of E-cadherin, such as ZEB1 and ZEB2 [14], [15]. The balance between these intrinsic regulators, including both transcription factors and miRNAs, is controlled by extrinsic signals, such as soluble mediators from the tumor microenvironment. Transforming growth factor beta (TGF-β), which is implicated in various tumor metastases, has been identified as the main factor involved in EMT in the tumor microenvironment [16], [17].

Interleukin-6 (IL-6) is another key factor in the tumor microenvironment, which is involved in tumorigenesis and progression [18], [19]. IL-6 activates the IL-6 receptor (IL-6R) to initiate signaling through the Janus kinase (JAK)/signal transducers and activators of transcription (STAT) signaling pathway and also NF-κB [20]. Elevated levels of IL-6 correlate with poor prognosis for a number of types of cancer, such as breast cancer and lung cancer [21], [22]. Recently, some research groups have reported that IL-6 contributes to tumor metastasis and EMT in breast cancer and ovarian cancer via the JAK/STAT3 signaling pathway [22], [23]. However, the role of IL-6 during the EMT process in lung adenocarcinoma remains poorly defined.

Metformin, an anti-diabetic drug, is associated with a reduced risk of developing many types of cancer [24], [25]. Studies have found that treatment of type 2 diabetics with metformin resulted in reduced cancer incidence and improved survival [26], [27]. Several pharmacological mechanisms may be involved in the anti-tumor function of metformin. Previous studies have found that metformin could inhibit the expression of pro-inflammatory mediators, such as IL-6 and IL-17, which play important roles in tumor development, by reducing activation of NF-κB [28], [29]. Moreover, metformin could inhibit cell growth and induce apoptosis in triple-negative breast cancers by blocking STAT3 phosphorylation [30]. Thirdly, recent studies have shown that metformin could regulate breast cancer stem cell EMT and significantly down regulate the expression of several EMT markers through decreasing the expression of key drivers of the EMT machinery, such as the transcription factors ZEB1, TWIST1 and Slug [31]. Since IL-6, through JAK/STAT3 signaling, might be involved in EMT in lung adenocarcinoma, and metformin can inhibit EMT and STAT3 phosphorylation in other cancer types, we hypothesized that metformin might be able to inhibit IL-6-induced EMT and growth and metastasis in lung adenocarcinoma.

In this study we provide novel evidence that enhanced IL-6 expression, via STAT3 phosphorylation, is a mechanism that can drive EMT and metastasis in lung adenocarcinoma. Metformin can inhibit IL-6 induced EMT by blocking STAT3 phosphorylation, suggesting a potential clinical use of metformin in the treatment of lung adenocarcinoma.

## Materials and Methods

### Cell lines and patient samples

Human lung adenocarcinoma cell lines A549 and HCC827 used in this study were purchased from the American Type Culture Collection and were maintained in RPMI 1640 with 10% FBS (Life Technologies, Inc.), 2 mmol/L of l-glutamine (Life Technologies), 100 µg/mL of streptomycin, and 100 units/mL of penicillin in a humidified 5% CO_2_ atmosphere at 37°C. HCC827-pSB388 cells stably over-expressing recombinant human IL-6 were established by infection of HCC827 cells with lentivirus pSB388, and further selected by puromycin at a concentration of 2 µg/mL, while the control cell line HCC827-pLVT7 was established by infection with lentivirus pLVT7. These cell lines were tested by using short tandem repeat profiling in September, 2012.

Lung adenocarcinoma and corresponding paracancerous tissues from patients treated at the Department of Respiratory Medicine, Daping hospital (Chongqing, China) from Aug 2011 to Sep 2012, were collected by surgical resection. This study was approved by the Institutional Review Board of the Third Military Medical University, China, and prior written and informed consent was obtained from every patient.

### Antibodies and drugs

Rabbit monoclonal antibodies against E-cadherin, vimentin, snail and phospho-Stat3 (Tyr705) were purchased from Cell Signaling Technology (Cambridge, MA, USA). Mouse monoclonal antibodies against β-actin and Peroxidase-Conjugated Affinipure goat anti-mouse IgG (H+L) were purchased from ZSBio (Beijing, China). Recombinant human IL-6 was produced by PeproTech (Rocky Hill, NJ). Metformin (Sigma, St. Louis, Missouri, USA) was dissolved in medium and stored at −20°C.

### Invasion assay

For the matrigel invasion assay, filters (8 µm pore size, Corning Costar Corporation) were precoated with 30 µl Matrigel (BD Biosciences, USA) for 3 h. Cells were starved in serum-free medium overnight, then trypsinized, centrifuged, and resuspended in serum-free RPMI 1640, and placed in the upper wells with 2×10^4^ cells per well supplemented with various concentrations of IL-6 (50, 100 and 500 ng/mL), which was replaced every other day. To determine the effect of metformin on cell invasion, various concentrations (1, 5, and 10 mmol/L) of metformin were added to the culture medium containing 50 ng/ml of IL-6. The lower wells of the transwells contained the same medium as the upper well but with the addition of 2% FBS. After incubation for 18 h, the cells on the upper well and the matrigel were gently removed with a cotton swab, and the cells on the bottom filter were fixed with 4% cold formaldehyde and stained with 0.1% crystal violet. The cells that were attached to the lower surface of the polycarbonate filter were counted under a light microscope (magnification, × 200). Each experiment was repeated three times.

### MTT assay

A total of 5000 cells (HCC827-pSB388 and HCC827 cells) were plated in 100 µl medium in 96-well plates. After 48 h incubation, various concentrations of metformin (2.5, 5, 7.5, 10 and 12.5 mmol/L) were added to each well, and cells were further cultured for 48 h. Then 10 µl of 5 mg/ml MTT (Sigma, St. Louis, Missouri, USA) in 100 µl medium was added to each well. After incubation for 4 h, medium was removed and 150 µl of DMSO was added to each well to dissolve the formazan crystals. Then the absorbance at 490 nm was determined using a ThermoFisher Spectrophotometer 1510 (Molecular Devices, Inc.). Cell viability was determined by dividing the absorbance values of treated cells to those of untreated cells.

### Immunofluorescence and immunohistochemistry

Immunofluorescence analysis was performed on 8-µm-thick frozen sections, or treated cells. Cells were cultured in 6-well chamber-slides, treated with IL-6 alone or with metformin for the indicated time periods, fixed with ice-cold 4% formaldehyde for 15 minutes at 37°C, and then blocked with rabbit serum for 20 minutes at room temperature before incubation with primary antibodies (E-cadherin 1∶200, vimentin 1∶50) overnight in the dark at 4°C. After three washes, slides were stained with PE-labeled secondary anti-rabbit antibody (1∶500) for 1 h at room temperature and nuclei were counterstained with DAPI. Stained cells were visualized with an Olympus confocal microscope.

Tumor specimens were fixed in 10% formalin, embedded in paraffin, and cut into 6 µm-thick sections. For immunohistochemistry, the sections were deparaffinized, rehydrated, incubated with 3% H_2_O_2_ for 10 min, and subjected to heat-induced antigen retrieval by boiling for 10 min in 0.01 M sodium citrate. The specimens were then blocked with normal goat serum for 1 hour and incubated overnight at 4°C with anti-E-cadherin (1∶400), anti-vimentin (1∶100) or anti-phospho-Stat3 (p-Tyr705) antibody (1∶400). The immune complexes were visualized using a kit obtained from the Beyotime Institute of Biotechnology (China) according to the manufacturer's instructions.

### Enzyme-linked immunosorbent array (ELISA)

STAT3-pY705 ELISA kit was purchased from Invitrogen Corporation (Invitrogen Corporation, Camarillo, USA). Human IL-6 ELISA Kit was purchased from USCN Life Science Inc (Texas, USA). The levels of STAT3-pY705 and IL-6 in tissue lysates were measured according to the manufacturers' protocols. Absorbance at 450 nm was measured using a microplate reader (Bio-Rad, Hercules, CA, USA). Each measurement was performed in triplicate.

### Reverse transcription (RT)-real-time-PCR

Total RNA from cells and tissues was extracted using Trizol Reagent (Invitrogen, Carlsbad, California, USA) according to the manufacturer's protocols, and then reverse-transcribed using random hexamers to generate cDNA. Real-time PCR was carried out in 25 µL reactions with 10 pmol primers and the expression levels of E-cadherin, vimentin, snail and IL-6 were detected by real-time PCR using the ABI prism 7300 sequence detection system (Applied Biosystems, New York, USA). Experiments were performed three times in triplicate. The relative mRNA expression levels of E-cadherin, vimentin, snail, and IL-6 were calculated using the comparative Ct (ΔΔCt) method, with GAPDH as a reference gene. The gene-specific primer sequences were shown in Table S1.

### Western blot analysis

Cells were lysed with ice-cold M-PER Mammalian Protein Extraction Reagent (Thermo Fisher Scientific Inc, USA) containing PMSF and phosphatase inhibitors for 30 min on ice and then sonicated for 15 seconds to ensure complete cell lysis. Total cellular protein was separated using 8% SDS-PAGE and transferred to polyvinylidene fluoride membranes (Immobilon Transfer Membranes; Millipore). The membranes were blocked with 5% non-fat milk for 1 h, and then incubated with antibodies against p-Stat3 (Tyr705) (1∶2,000), total Stat3 (1∶2,000), E-cadherin (1∶1,000), vimentin (1∶1,000), snail (1∶1,000) and β-actin (1∶2,000) at 4°C overnight. The membrane was then washed three times with 0.1% Tween 20-TBS, and incubated with a horseradish peroxidase-linked secondary antibody (1∶2,000) for 1 h at room temperature. Bands were visualized via enhanced chemiluminescence (ECL) according to the manufacturer's instructions.

### In vivo xenograft experiments

A total of 18 six-week-old nude mice (half male and half female) were purchased from the Chinese Academy of Medical Sciences (Beijing, China) and housed and maintained in laminar flow cabinets under specific pathogen-free conditions. These mice were divided into three groups with 6 mice (3 male and 3 female) in each group: group 1 (HCC827) and group 2 (HCC827-pSB388) received pure drinking water, while group 3 (HCC827-pSB388+Met) received drinking water with metformin (250 mg/kg body weight) from 2 days before tumor cell inoculation until the mice were sacrificed. 2×10^6^ HCC827 (group 1) and HCC827-pSB388 (groups 2 and 3) lung cancer cells were injected subcutaneously. Engrafted mice were inspected biweekly for tumor appearance by visual observation and palpation. Tumor volume (mm^3^) was calculated as length × width^2^/2. Mouse care and use was performed in strict accordance with the ethical guidelines of the Third Military Medical University. The protocol was approved by the Laboratory Animal Welfare and Ethics Committee of the Third Military Medical University (Permit Number: SYXK-PLA-2007035). Mice were CO_2_ -euthanized, and all efforts were made to minimize suffering.

### Statistical analysis

All the data from quantitative assays were expressed as the mean ± standard deviation. Statistical analyses were performed using the independent-samples t-test or one-way ANOVA. The difference was considered statistically significant when *p*<0.05. All statistical analyses were carried out with SPSS 18.0 software (Chicago, USA).

## Results

### IL-6 induces EMT and enhances tumor cell invasion in vitro

**Figure 1:**
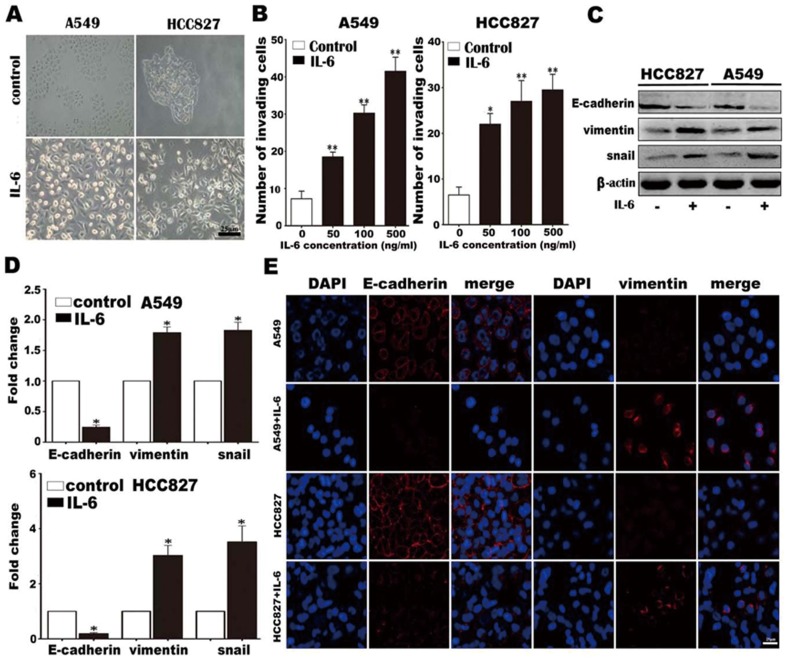
IL-6 promotes lung carcinoma cell invasion and EMT. (A) A549 and HCC827 cell mesenchymal phenotype induced by IL-6 (200×). (B) A549 and HCC827 cell invasion in the presence of various concentrations of IL-6. (C) E-cadherin, vimentin and snail protein levels were examined by Western blotting. Equal protein loading was verified by β-actin. (D) mRNA expression levels of E-cadherin, vimentin and snail were analyzed by quantitative PCR and normalized by GAPDH. (E) E-cadherin and vimentin protein levels were detected by immunofluorescence (800×). Error bars represent the standard deviation. *, p<0.05, **, p<0.005.

To determine whether IL-6 is involved in EMT, we first examined the effect of IL-6 on the morphology of lung adenocarcinoma cells. In RPMI 1640 medium, A549 and HCC827 cells exhibited features typical of epithelial cells, displaying a distinctive cobblestone appearance and forming clusters. However, after stimulation with 100 ng/mL IL-6 for 1 week, both A549 and HCC827 cells became scattered and acquired fibroblast-like shapes, which are characteristics of mesenchymal-like morphology (Fig. 1A). These changes in morphology suggest that A549 and HCC827 cells might undergo EMT in response to IL-6 stimulation. It has been established that mesenchymal cells are endowed with enhanced invasion and metastatic capabilities. Therefore, we determined the effect of IL-6 on the cells' invasion abilities. The results showed that IL-6 treatment could significantly enhance invasive capacity in a dose-dependent manner for both A549 and HCC827 cell lines (Fig. 1B and Fig. S1). Importantly, Western blotting (Fig. 1C), quantitative PCR (Fig. 1D) and immunofluorescence (Fig. 1E) showed that treatment of both A549 and HCC827 cells with 50 ng/mL IL-6 for 1 week reduced the expression of an epithelial cell marker (E-cadherin), and increased the expression of mesenchymal cell markers (vimentin and snail). Collectively, the alterations in cell morphology, increased invasive capacity, acquisition of mesenchymal cell markers and loss of epithelial cell markers indicate that lung adenocarcinoma cells undergo EMT upon IL-6 stimulation.

### A negative correlation between IL-6 production and E-cadherin expression, and a positive correlation between IL-6 production and vimentin expression are detectible in lung adenocarcinoma tissues

**Figure 2:**
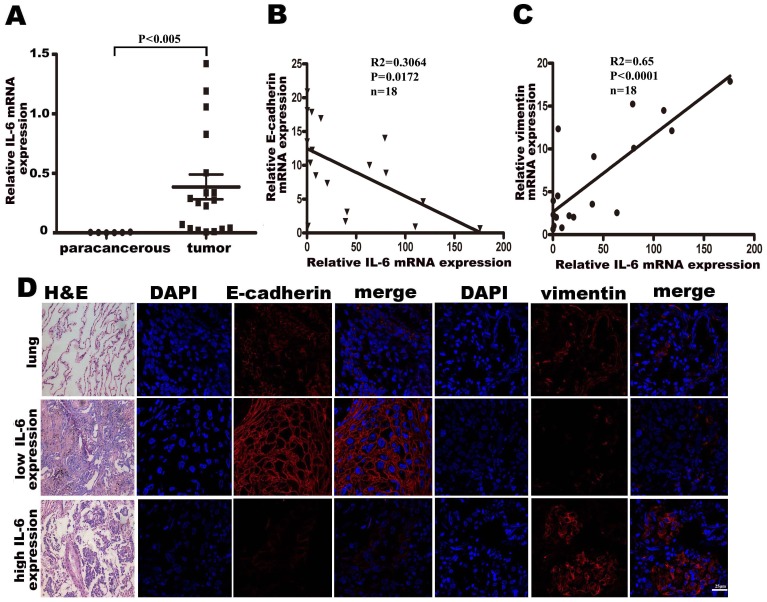
Correlation between IL-6 and E-cadherin or vimentin in patient samples. (A) IL-6 gene expression levels in tumor (n = 18) and corresponding paracancerous (n = 6) tissues was analyzed by real-time PCR, with GAPDH as a reference gene. p<0.005. (B) IL-6 and E-cadherin gene expression in tumor tissues was analyzed by real-time PCR and the correlation between these two genes was analyzed. p<0.05. (C) IL-6 and vimentin gene expression in tumor tissues was analyzed by real-time PCR and the correlation between these two genes was analyzed (p<0.0001). (D) Representative immunofluorescence staining for E-cadherin and vimentin in lung adenocarcinoma tissues with low IL-6 or high IL-6 expression (800×).

As we demonstrated that stimulation with IL-6 in vitro could induce EMT in lung adenocarcinoma cell lines, we speculate that there may be a correlation between IL-6 production and mesenchymal or epithelial cell markers in lung adenocarcinoma tissues. Accordingly, we examined the expression of IL-6, E-cadherin and vimentin mRNA in tissue samples from 18 cases of lung adenocarcinoma, and 6 corresponding paracancerous tissues, by quantitative PCR, and analyzed the correlation between IL-6 and E-cadherin or vimentin expression. As expected, we found that IL-6 mRNA expression in lung adenocarcinoma tissues was much higher than in paracancerous tissues (p<0.005, Fig. 2A), similar to results reported previously [21], [22]. Importantly, there was a negative correlation between IL-6 and E-cadherin expression (Fig. 2B), and a positive correlation between IL-6 and vimentin expression (Fig. 2C). We also used immunofluorescence assays to determine the protein levels of E-cadherin and vimentin in lung adenocarcinoma tissue samples with low IL-6 or high IL-6 expression. The results showed weak vimentin expression but strong E-cadherin expression in low IL-6-expressing cancer tissues, whereas strong vimentin expression but weak E-cadherin expression was observed in high IL-6-expressing cancer tissues (Fig. 2D). The correlation between IL-6 and EMT markers in clinical samples, together with the in vitro IL-6 stimulation results, strongly suggests that IL-6 is involved in EMT in lung adenocarcinoma.

### IL-6 activates STAT3 phosphorylation in vitro and a positive correlation between IL-6 production and STAT3 phosphorylation is detected in lung adenocarcinoma tissues

**Figure 3:**
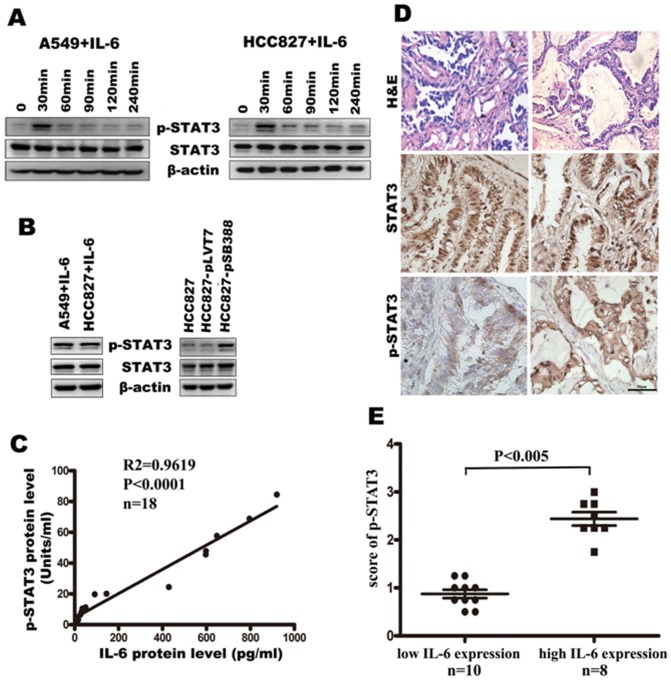
IL-6 induces STAT3 phosphorylation in A549 and HCC827 cells in vitro and IL-6 expression is positively correlated with STAT3 phosphorylation in patient tissure samples. (A) STAT3 phosphorylation status in A549 and HCC827 cells after stimulation with IL-6 in a short time. (B) STAT3 phosphorylation status was determined by western blotting. A549 and HCC827 cells were stimulated with IL-6 in a long time (left). HCC827-pSB388 cells were stimulated with IL-6. HCC827 and HCC827-pLVT7 cells were used as a control (right). (C) Correlation between IL-6 production and STAT3 phosphorylation in human lung adenocarcinoma tissues (n = 18, p<0.0001). (D) Representative immunohistochemical staining for STAT3 phosphorylation in lung adenocarcinoma tissues with low IL-6 or high IL-6 expression (400 ×). (E) Comparison of the score of p-STAT3 with different IL-6 expression level.

The involvement of the JAK-STATs signaling pathway in IL-6/IL-6R signaling and involvement of STAT3 phosphorylation in EMT of cancer cells has been proved by lots of results [20], [32], [33]. Therefore, we speculate that the IL-6-induced EMT of lung adenocarcinoma cells may be due to activation of STAT3. To investigate this point, we first examined the effect of IL-6 on STAT3 tyrosine phosphorylation in A549 and HCC827 lung adenocarcinoma cells. As expected, we found that 50 ng/mL IL-6 activated STAT3 and induced STAT3 tyrosine phosphorylation on Tyr 705 in A549 and HCC827 cells. STAT3 was rapidly phosphorylated upon IL-6 treatment, reaching the highest level at the 30 minute time point, and then declined to the basal level at 120 minutes (Fig. 3A). We also detected the STAT3 phosphorylation in A549 and HCC827 cells treated with 50 ng/mL of IL-6 for 1 week. The results showed that high STAT3 phosphorylation level was detected after IL-16 treatment for 1 week (left panel, Fig. 3B). Moreover, we detected STAT3 phosphorylation in HCC827-pSB388 cells, which stably over expressed IL-6. Consistent with IL-6 stimulation, HCC827-pSB388 cells, but not HCC827 or HCC827-pLVT7 cells, displayed STAT3 phosphorylation (right panel, Fig. 3B). Importantly, the level of IL-6 was positively associated with the level of STAT3 phosphorylation in lung adenocarcinoma tissues when the values for each individual patient were plotted (p<0.005, Fig. 3C). Moreover, we found significantly higher STAT3 phosphorylation in high IL-6-producing tumor tissues than that in low IL-6-producing tissues (Fig. 3D–E). Again, these data provide strong support for our hypothesis that induction of EMT by IL-6 is through activation of STAT3.

### Metformin inhibits IL-6-induced EMT in lung adenocarcinoma cells

**Figure 4:**
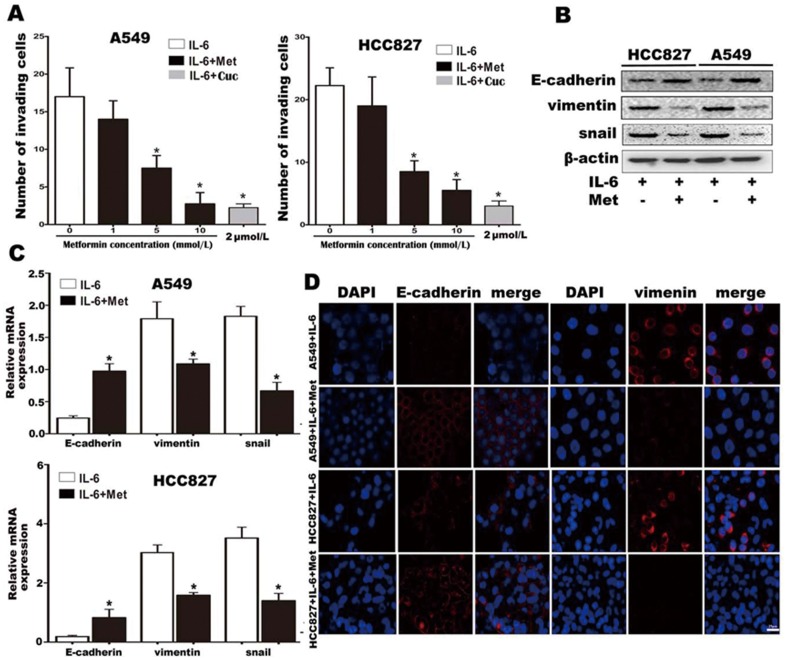
Metformin inhibits IL-6 promotion of lung carcinoma cell invasion and EMT in vitro. (A) Invasion of IL-6 treated lung cancer cells in the presence of various concentrations of metformin. The selective STAT3 inhibitor cucurbitacin Q (Cuc, 2 µmol/L) served as a positive control for inhibition of lung cancer cell invasion. (B) Protein expression levels of E-cadherin, vimentin and snail in cells treated with IL-6 alone or in combination with metformin were analyzed by western blotting. β-actin was used as a loading control. (C) mRNA expression levels of E-cadherin, vimentin and snail in cells treated with IL-6 alone or in combination with metformin were examined by real time PCR and normalized by GAPDH. (D) The expression of E-cadherin and vimentin was detected by immunofluorescence in A549 and HCC827 cells treated with IL-6 alone or in combination with metformin (800×). Error bars represent the standard deviation (*, p<0.05; **, p<0.005).

Since TGF-β is involved in EMT, TGF-β-neutralizing antibodies and TGF-β receptor inhibitors have been employed in attempts to inhibit EMT, thereby reducing tumor metastasis and enhancing chemotherapy sensitivity [34], [35]. Our discovery of the involvement of IL-6 in the induction of EMT provides an impetus to find a drug to block IL-6-induced EMT. Previous studies have shown that metformin could inhibit STAT3 phosphorylation, which is involved in the IL-6/IL-6R signaling pathway and, as we have shown, IL-6-induced EMT. Therefore, we hypothesize that metformin might be a potential drug to inhibit IL-6-induced EMT. To investigate this, we first determined the effect of metformin on IL-6-induced EMT in vitro. Consistent with our hypothesis, our data revealed that 5 mmol/L (p<0.05) or 10 mmol/L (p<0.005) metformin could significantly reduce lung cancer cell invasion, which was induced by IL-6 (Fig. 4A and Fig. S2) in both A549 and HCC827 cell lines. The extent of metformin inhibition of invasion at 10 mmol/L was similar to that seen with 2 µmol/L cucurbitacin Q, a specific STAT3 inhibitor (Fig. 4A). Additionally, quantitative PCR (Fig. 4C), Western blotting (Fig. 4B) and immunofluorescence (Fig. 4D) showed that metformin could reverse the IL-6 treatment-induced changes, significantly repressing vimentin and snail expression, and restoring E-cadherin expression. Collectively, these in vitro results showed that metformin inhibits IL-6-induced EMT in lung adenocarcinoma cells.

### Metformin suppresses IL-6-enhanced tumor growth and metastasis in nude mice

To examine the potential role of metformin in tumor growth and metastasis of lung adenocarcinoma, we first established stable IL-6-expressing HCC827 cells (HCC827-pSB388) by lentivirus infection. This was necessary since IL-6 in the tumor tissues is mainly produced by infiltrating inflammatory cells and, being of mouse origin, this IL-6 will have no effect on human tumor cells. We then examined the expression of E-cadherin and vimentin protein in HCC827-pLVT7 and HCC827-pSB388 cells by Western blotting, and the cells' invasive capacity. The results showed that the over-expression of IL-6 in HCC827-pSB388 cells could repress E-cadherin expression and elevate vimentin expression, and also enhanced the cells' invasive capacity in vitro (Fig. S3). These results were consistent with the results from the IL-6 stimulation experiments.

**Figure 5:**
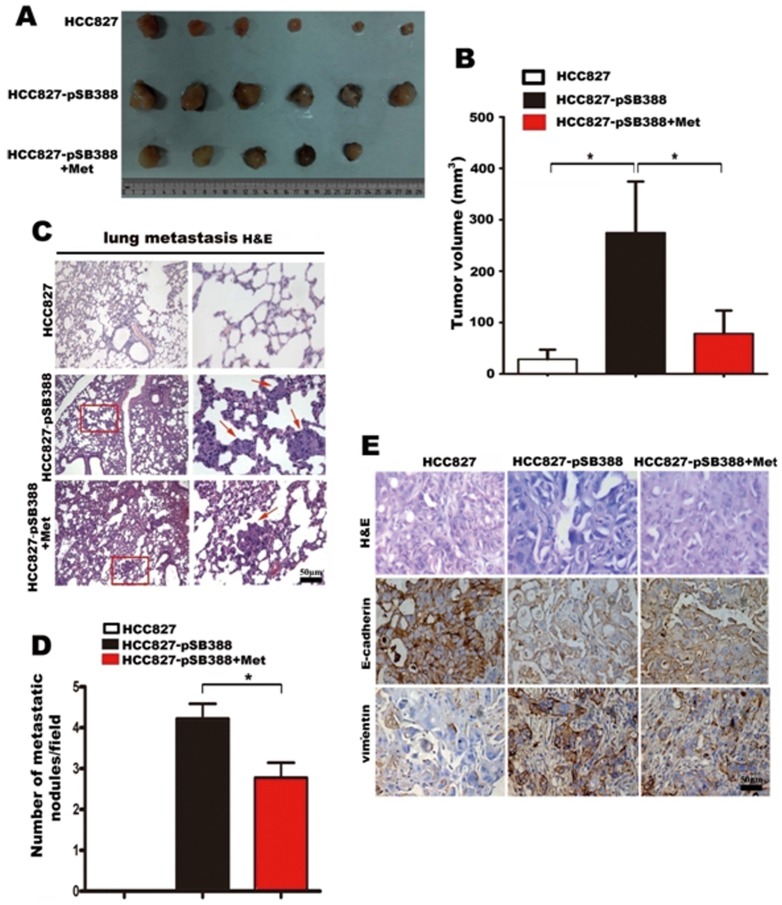
Metformin inhibits tumor growth, EMT, and metastasis induced by IL-6 in vivo. (A) Xenograft at sacrifice. Xenografts from HCC827-pSB388 group were much larger than HCC827 group. Xenografts from HCC827-pSB388+Met group, which treated with metformin, were much smaller than that from HCC827-pSB388 group. (B) Tumor volumes were determined at the time of sacrifice. (C) Metastatic tumor nodules in the lung were examined by H & E staining of serial sections. Tumor nodules are marked with red arrows (100× and 400×). (D) The numbers of cancerous metastatic nodules in these lung sections were counted and the average number per field of view is presented. (E) E-cadherin and vimentin expression in tumor tissues from HCC827, HCC827pSB388 and HCC827pSB388+Met groups was analyzed by immunohistochemistry (400×). Error bars represent the standard deviation (*, p<0.05).

We next investigated the effect of IL-6 expression on tumor growth and metastasis in vivo using xenograft experiments. We found that IL-6 transfection significantly promoted the growth of HCC827 tumors (Fig. 5A–B), consistent with the previous findings of a pro-tumor activity of IL-6 in lung adenocarcinoma [36]. However, this enhancement of tumor growth by IL-6 transfection was partially reversed by metformin treatment (Fig. 5A–B), indicating an inhibitory effect of metformin on IL-6-induced tumor growth. Importantly, we found significantly fewer metastatic nodes in lung and liver tissues from metformin-treated HCC827-pSB388-bearing mice than in PBS-treated HCC827-pSB388-bearing mice, whereas there was no metastatic node from HCC827-bearing mice (p<0.05) (Fig. 5C–D). In addition, using MTT, we found that metformin significantly decreased the proliferation of both HCC827 cells and HCC827-pSB388 cells (Fig. S4).

Given the in vitro evidence that metformin could inhibit EMT of lung adenocarcinoma cells, we suppose that the reduction in metastasis formation seen with metformin was due to inhibition of HCC827-pSB388 cell EMT. Consequently, we examined the expression of EMT markers in HCC827, HCC827-pSB388 and metformin-treated HCC827-pSB388 tumor tissues with immunohistochemistry. As expected, we found reduced E-cadherin expression and enhanced vimentin expression in HCC827-pSB388 tumors compared with HCC827 tumors, further supporting the in vitro findings that IL-6 could induce EMT of lung adenocarcinoma cells. Interestingly, metformin could partially decrease EMT caused by IL-6 over expression (Fig. 5E). These results suggest that metformin inhibits tumor growth and metastasis of lung adenocarcinoma cells in xenografted mice via inhibition of EMT.

### Metformin inhibits lung adenocarcinoma EMT through inhibition of IL-6-induced STAT3 tyrosine phosphorylation

**Figure 6:**
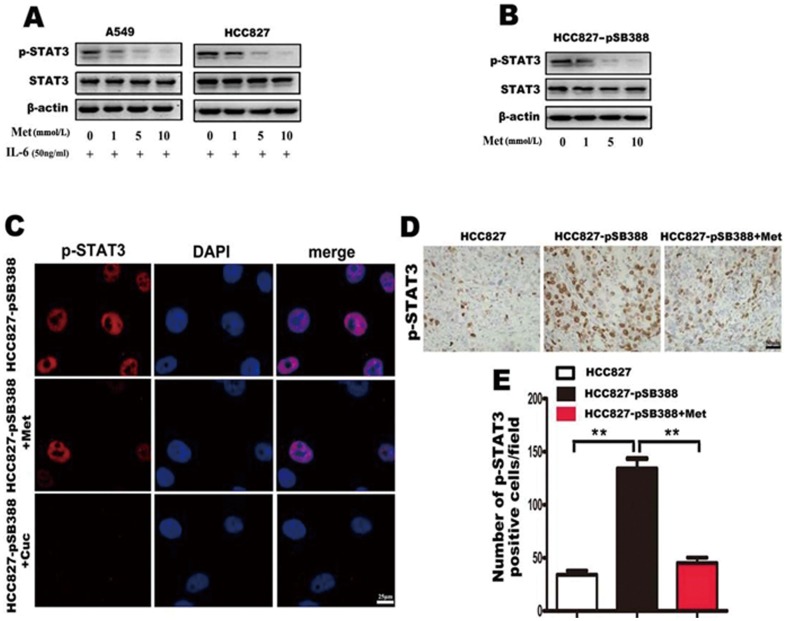
Metformin suppresses IL-6-induced STAT3 phosphorylation in lung adenocarcinoma cells. (A) Lung cancer cells were treated with IL-6 and the indicated concentrations of metformin, STAT3 phosphorylation status was determined by western blotting. Metformin suppressed STAT3 phosphorylation induced by IL-6 in lung adenocarcinoma cells in a dose-dependent manner. (B) CC827-pSB388 cells were treated with the indicated concentrations of metformin and STAT3 phosphorylation was determined by western blotting. (C) STAT3 phosphorylation in untreated HCC827-pSB388 cells, HCC827-pSB388 cells treated with metformin and HCC827-pSB388 cells treated with cucurbitacin Q were examined by immunofluorescence (800×). (D) STAT3 phosphorylation in tumors from HCC827, HCC827-pSB388 and HCC827-pSB388+Met groups was examined by immunohistochemistry (400×). (E) The average number of p-STAT3 positive cells in each field of view was analyzed. Error bars represent the standard deviation (**, p<0.005).

To elucidate the underlying mechanisms of metformin inhibition of IL-6-induced EMT, we first determined the effect of metformin on IL-6-induced STAT3 tyrosine phosphorylation. We added 1–10 mmol/L metformin to A549 and HCC827 cells cultured in medium containing 50 ng/mL of IL-6, and to HCC827-pSB388 cells for 1 week. The results showed that metformin treatment could significantly reduce IL-6-induced STAT3 phosphorylation in A549, HCC827 and HCC827-pSB388 lung adenocarcinoma cells in a dose-dependent manner (Fig. 6A-B). Immunofluorescence assay on HCC827-pSB388 cells further showed that metformin inhibits STAT-3 phosphorylation and the effect is similar to a specific STAT3 inhibitor, cucurbitacin Q (Fig. 6C). These results suggest that metformin inhibits EMT of lung adenocarcinoma cells through inhibition of IL-6-induced STAT3 tyrosine phosphorylation in vitro. Next we examined STAT3 phosphorylation in tumors from HCC827 and HCC827-pSB388 cell grafted mice by immunohistochemistry. We found more STAT3 phosphorylation in tumors from HCC827-pSB388 cell grafted mice than in tumors from HCC827 cells grafted mice (Fig. 6D-E, p<0.001). This result was consistent with the findings from clinical samples, which showed a positive correlation between IL-6 production and STAT3 phosphorylation (Fig. 3C–E). However, the phosphorylation of STAT3 was significantly reduced in tumors from HCC827-pSB388 cell grafted mice that were treated with metformin (Fig. 6D–E). These results suggest that inhibition of STAT3 phosphorylation might be the underlying mechanism of metformin inhibition of IL-6-induced EMT.

Further, to determine the effect of metformin on AMPK activation in HCC827 cells and HCC827-pSB388 cells, Western blot analysis was performed. As shown in Fig. S5, Western Blot results showed that expression of phosphorylated AMPK in HCC827-pSB388 cells was lower when compared to that of HCC827 cells. Metformin treatment significantly increased activation of AMPK in HCC827-pSB388 cells (Fig. S5).

## Discussion

EMT is an important biological process that plays a critical role in tumorigenesis. However, the mechanisms underlying EMT in lung adenocarcinoma and how this process is inhibited remain to be explored. In this study, we showed that IL-6 was involved in EMT and metastasis, via induction of STAT3 phosphorylation in lung adenocarcinoma cells, by in vitro IL-6 stimulation experiments, clinical correlation analysis and xenograft experiments. In addition, we found that metformin could inhibit IL-6 enhancement of tumor growth, as well as reduce tumor metastasis, through inhibition of STAT3-mediated EMT. Collectively, these results not only suggest that the IL-6/IL-6R/STAT3 may be used as molecular targets for the inhibition of IL-6-induced EMT, but also provide experimental evidence for the potential use of metformin in the treatment of lung adenocarcinoma.

It is well known that inflammatory mediators and cells are involved in the migration, invasion and metastasis of malignant cells [37]. However, most studies into the mechanisms of inflammation-driven metastasis have focused on the induction of chemokine receptors, which can direct the movement of cancer cells, by inflammatory cytokines such as TNF-α, IL-1β and IL-6 [37], [38]. Growing evidence has suggested that inflammatory mediators, produced by tumor-infiltrating leukocytes and cancer-related fibroblasts, also promote tumor metastasis via inducing EMT [39]–[41]. Numerous reports indicate that growth factors, including TGF-β, epidermal growth factor (EGF), vascular endothelial growth factor, platelet-derived growth factor and hepatocyte growth factor can induce cancer cell EMT in a variety of cancers including lung adenocarcinoma [42]. Recently, another inflammatory cytokine IL-6 has been reported as a potent inducer of EMT in breast cancer cells with an epithelial phenotype [22]. Previous studies have found IL-6 in the serum of approximately 40% of patients with NSCLC [43], [44]. Koh and colleagues found that the expression of IL-6 in tumor tissues correlated with the concentration of serum IL-6, tumor progression, and the overall survival in NSCLC [45]. Taken together, these findings suggest a role for IL-6 in the progression of lung adenocarcinoma. In this study, we demonstrated that IL-6 could drive EMT and promote metastasis in lung adenocarcinoma, similar to the function of IL-6 in breast cancer and head and neck tumor metastasis.

Mesenchymal-like cancer cells in lung adenocarcinoma are endowed with enhanced invasive ability and resistance to chemotherapy and tyrosine kinase inhibitors (TKI) [46]. Therefore, pharmacologists are focusing on the identification of EMT inhibitors. Since the role of TGF-β in EMT was first recognized, TGF-β-neutralizing antibodies and TGF-β receptor inhibitors have been studied to inhibit EMT [34], [35]. Recently, metformin, which is widely used as first-line drug for type 2 diabetes, is found to repress TGF-β-induced EMT in breast cancer [47]. However, whether metformin could also inhibit IL-6-induced EMT remains to be explored. In this study, we found that metformin could not only retard IL-6 enhancement of tumor growth, but also reduce tumor metastasis in lung adenocarcinoma cell-bearing nude mice. Importantly, we found that metformin partially inhibited IL-6-induced EMT and induced the reacquisition of an epithelial phenotype as characterized by the gain of epithelial marker expression and decreased expression of mesenchymal markers. To our knowledge, this was the first study that showed metformin could inhibit IL-6-mediated EMT and tumor metastasis. Interestingly, metformin inhibited tumor cell growth in vitro and tumor growth in vivo. Thus, the inhibitory effect of metformin on tumor metastasis might be due to the reduced number of tumor cells induced by metformin. We do not rule out this possibility. However, the relationship between the effect of metformin on tumor metastasis and the effect of metformin on tumor growth still needs further investigation.

IL-6/IL-6R performs biological functions mainly through JAK-STAT3. It has been reported that the activation of STAT3 in tumor cells promotes tumor growth by increasing the capacity of tumors to evade the immune system [48], [49]. Additionally, STAT3 is involved in EMT in ovarian cancer, and STAT3 phosphorylation is significantly correlated with TNM (tumor, lymph node, and metastasis stages) [50]. In addition, previous results have revealed metformin could block STAT3 phosphorylation in triple-negative breast cancers [30]. In this study, we found that metformin treatment inhibited STAT3 phosphorylation and induced AMPK phosphorylation in HCC827 cells and HCC827-pSB388 cells. It is reported that activated AMPK can suppress STAT3 signaling and STAT3 phosphorylation [51], [52]. Although metformin may directly target STAT3 [30], it is still possible that the effect of metformin on STAT3 phosphorylation may be indirectly mediated through AMPK activation. However, the direct effect and mechanism of AMPK activation induced by metformin on STAT3 in lung cancer cells still needs further investigation. Based on the knowledge mentioned above and our new findings that metformin repressed the IL-6-activated STAT3 phosphorylation, we suggest that metformin inhibits IL-6-induced EMT and metastasis of lung adenocarcinoma cells via blockade of STAT3 phosphorylation. However, we cannot exclude the possibility of other potential mechanisms contributing to the inhibition of IL-6-induced EMT by metformin.

In conclusion, we demonstrated that enhanced IL-6 production, via STAT3 phosphorylation, was one of the underlying mechanisms of EMT and metastasis in lung adenocarcinoma. We found that metformin could inhibit IL-6 induced EMT possibly by blocking STAT3 phosphorylation, suggesting a potential clinical use of metformin in treatment of lung adenocarcinoma.

## Supporting Information

Figure S1 **IL-6 promoted A549 and HCC827 cells invasion.** A549 and HCC827 cells invasion was promoted by various concentrations IL-6 (200×).
(TIF)

Figure S2 **Metformin inhibited A549 and HCC827 cells invasion.** Metformin inhibited A549 and HCC827 cells invasion, which was induced by 50 ng/ml IL-6 at the various concentrations. As a positive control, Cuc (2 µmol/L), a p-STAT3 special inhibitor, inhibited A549 and HCC827 cells invasion (200×).
(TIF)

Figure S3 **Overexpressed IL-6 promoted HCC827 cell invasion and EMT.** (A) Over-expression of IL-6 was verified by western blotting. No IL-6 expression was detected in the control cell line HCC827 and the negative control cell HCC827-pLVT7. There was a strong IL-6 expression in the IL-6 overexpression cell HCC827-pSB388. GAPDH was used as a loading control. (B) Over-expression of IL-6 promoted invasion of HCC827 cells (400×). (C) The numbers of the invasion cells were statistically analyzed. Error bars represent the standard deviation (*, p<0.05). (D) The E-cadherin and vimentin expression in cell HCC827-pSB388 was analyzed by western blotting. Compared with HCC827 and HCC827-pLVT7, overexpression of IL-6 repressed E-cadherin expression and elevated vimentin expression. β-actin was used as a loading control.
(TIF)

Figure S4 **Metformin decreased proliferation of both HCC827 cells and HCC827-pSB388 cells.** Cells were untreated or treated with different doses of metformin as indicated for 48 hours. Cell viability was assayed using MTT method. Data was expressed as Mean ± SEM. *, p<0.05, #, p<0.01.
(TIF)

Figure S5 **Metformin activation of AMPK in HCC827-pSB388 cells.** Expression of phosphoralylated AMPK and total AMPK were detected using Western blot analysis in HCC827 cells and HCC827-pSB388 cells, untreated or treated with metformin. β-actin was used as a loading control.
(TIF)

Table S1 (DOC)
