# Supplementary figures and images for: Metformin Inhibits the IL-6-Induced Epithelial-Mesenchymal Transition and Lung Adenocarcinoma Growth and Metastasis
Source: PLoS One. 2014 Apr 30;9(4):e95884. doi: 10.1371/journal.pone.0095884 (PMC4005743; doi:10.1371/journal.pone.0095884)

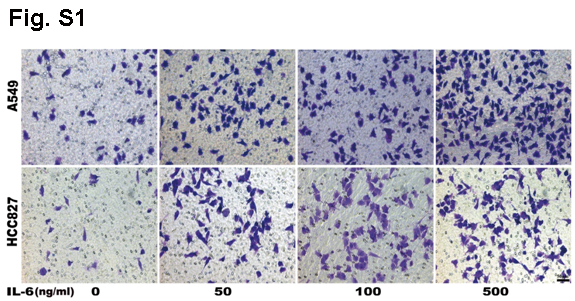

Supplement: Figure S1 — IL-6 promoted A549 and HCC827 cells invasion. A549 and HCC827 cells invasion was promoted by various concentrations IL-6 (200×). (TIF) [file pone.0095884.s001.tif]

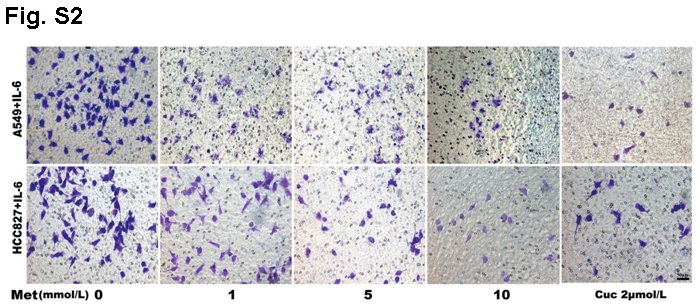

Supplement: Figure S2 — Metformin inhibited A549 and HCC827 cells invasion. Metformin inhibited A549 and HCC827 cells invasion, which was induced by 50 ng/ml IL-6 at the various concentrations. As a positive control, Cuc (2 µmol/L), a p-STAT3 special inhibitor, inhibited A549 and HCC827 cells invasion (200×). (TIF) [file pone.0095884.s002.tif]

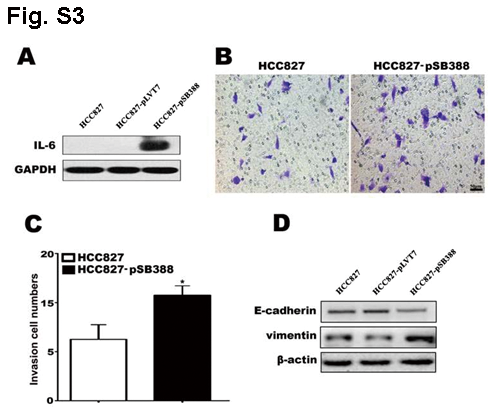

Supplement: Figure S3 — Overexpressed IL-6 promoted HCC827 cell invasion and EMT. (A) Over-expression of IL-6 was verified by western blotting. No IL-6 expression was detected in the control cell line HCC827 and the negative control cell HCC827-pLVT7. There was a strong IL-6 expression in the IL-6 overexpression cell HCC827-pSB388. GAPDH was used as a loading control. (B) Over-expression of IL-6 promoted invasion of HCC827 cells (400×). (C) The numbers of the invasion cells were statistically analyzed. Error bars represent the standard deviation (*, p<0.05). (D) The E-cadherin and vimentin expression in cell HCC827-pSB388 was analyzed by western blotting. Compared with HCC827 and HCC827-pLVT7, overexpression of IL-6 repressed E-cadherin expression and elevated vimentin expression. β-actin was used as a loading control. (TIF) [file pone.0095884.s003.tif]

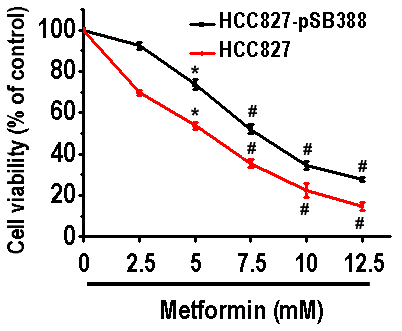

Supplement: Figure S4 — Metformin decreased proliferation of both HCC827 cells and HCC827-pSB388 cells. Cells were untreated or treated with different doses of metformin as indicated for 48 hours. Cell viability was assayed using MTT method. Data was expressed as Mean ± SEM. *, p<0.05, #, p<0.01. (TIF) [file pone.0095884.s004.tif]

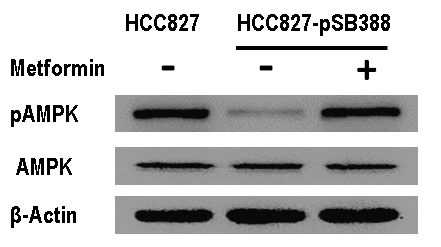

Supplement: Figure S5 — Metformin activation of AMPK in HCC827-pSB388 cells. Expression of phosphoralylated AMPK and total AMPK were detected using Western blot analysis in HCC827 cells and HCC827-pSB388 cells, untreated or treated with metformin. β-actin was used as a loading control. (TIF) [file pone.0095884.s005.tif]
