# Supplementary material for: Metformin Inhibits the IL-6-Induced Epithelial-Mesenchymal Transition and Lung Adenocarcinoma Growth and Metastasis
Source: PLoS One. 2014 Apr 30;9(4):e95884. doi: 10.1371/journal.pone.0095884 (PMC4005743; doi:10.1371/journal.pone.0095884)
Supplement: Table S1 — (DOC) [file pone.0095884.s006.doc]

**Table S1.**

| gene | sense | antisense |
| --- | --- | --- |
| E-cadherin | 5′-AAGGAGGCGGAGAAGAGGAC-3′ | 5′-CGTCGTTACGAGTCACTTCAGG-3′ |
| Vimentin | 5′- AAGAGAACTTTGCCGTGGAA-3′ | 5′-TCCAGCAGCTTCCTGTAGGT-3′ |
| Snail | 5′-GAGGCGGTGGCAGACTAG-3′ | 5′-GACACATCGGTCAGACCAG-3′ |
| IL-6 | 5’-CTTTTGGAGTTTGAGGTAGTATACCTA-3’ | 5’-GCTGCGCAGAATGAGATGAGTTGTC-3’ |
| GAPDH | 5′-CCCTCAAGATTGTCAGCAATGC-3′ | 5′-GTCCTCATGTTAGCCCAGGAT-3′ |
